# Influence of Elevated Temperatures on Resistance Against Phoma Stem Canker in Oilseed Rape

**DOI:** 10.3389/fpls.2022.785804

**Published:** 2022-03-02

**Authors:** Katherine Noel, Aiming Qi, Lakshmi Harika Gajula, Craig Padley, Steffen Rietz, Yong-Ju Huang, Bruce D. L. Fitt, Henrik U. Stotz

**Affiliations:** ^1^Centre for Agriculture, Food and Environmental Management, School of Life and Medical Sciences, University of Hertfordshire, Hatfield, United Kingdom; ^2^LS Plant Breeding Ltd., Cambridge, United Kingdom; ^3^NPZ Innovation GmbH, Holtsee, Germany

**Keywords:** phoma stem canker, quantitative resistance, climate change, oilseed rape, temperature-sensitivity

## Abstract

Cultivar resistance is an important tool in controlling pathogen-related diseases in agricultural crops. As temperatures increase due to global warming, temperature-resilient disease resistance will play an important role in crop protection. However, the mechanisms behind the temperature-sensitivity of the disease resistance response are poorly understood in crop species and little is known about the effect of elevated temperatures on quantitative disease resistance. Here, we investigated the effect of temperature increase on the quantitative resistance of *Brassica napus* against *Leptosphaeria maculans*. Field experiments and controlled environment inoculation assays were done to determine the influence of temperature on *R* gene-mediated and quantitative resistance against *L. maculans*; of specific interest was the impact of high summer temperatures on the severity of phoma stem canker. Field experiments were run for three consecutive growing seasons at various sites in England and France using twelve winter oilseed rape breeding lines or cultivars with or without *R* genes and/or quantitative resistance. Stem inoculation assays were done under controlled environment conditions with four cultivars/breeding lines, using avirulent and virulent *L. maculans* isolates, to determine if an increase in ambient temperature reduces the efficacy of the resistance. High maximum June temperature was found to be related to phoma stem canker severity. No temperature effect on stem canker severity was found for the cultivar ES Astrid (with only quantitative resistance with no known *R* genes). However, in the controlled environmental conditions, the cultivar ES Astrid had significantly smaller amounts of necrotic tissue at 20°C than at 25°C. This suggests that, under a sustained temperature of 25°C, the efficacy of quantitative resistance is reduced. Findings from this study show that temperature-resilient quantitative resistance is currently available in some oilseed cultivars and that efficacy of quantitative resistance is maintained at increased temperature but not when these elevated temperatures are sustained for a long period.

## Introduction

The plant immune system consists of two branches: a primary basal defense response known as the pathogen-associated molecular pattern (PAMP)-triggered immunity (PTI), and a specific effector-triggered immune (ETI) response. PTI recognizes conserved molecules common to classes of microbes while ETI recognizes and responds to effectors produced by pathogens adapted to overcome PTI. Jones and Dangl (2006) originally proposed a Zigzag model to explain the strength and evolution of PTI and ETI; whereas PTI depends on the perception of PAMPs by pattern recognition receptors (PRRs), ETI involves effector recognition by nucleotide-binding leucine-rich repeat receptors (NLRs). Since this model was proposed, advances have been made in understanding plant immunity, revealing its limitations (Pritchard and Birch, 2014). It has recently become clear that PTI and ETI influence one another to generate a comprehensive immune response (Yuan et al., 2021). Further issues arose from the confusion over the classification of resistance against apoplastic pathogens, such as *Leptosphaeria maculans*, as PTI or ETI (Jones and Dangl, 2006; Thomma et al., 2011; Stotz et al., 2014). The classification of effector-triggered defense (ETD) as another form of resistance, in addition to ETI and PTI, was first proposed by Stotz et al. (2014). ETD replaces ETI when extracellular apoplastic pathogens are encountered. Involving different receptors (ETI is triggered by intracellular NLRs), ETD differs from ETI in several aspects. The ETD response is delayed relative to ETI, which is often associated with fast, hypersensitive host cell death. Furthermore, with ETD, the pathogen is not killed and may resume growth following the onset of host senescence, or if the host resistance response is otherwise compromised (Stotz et al., 2014). All these plant immune and defensive mechanisms are influenced by temperature changes (Cheng et al., 2013).

Increased temperature has been linked to more severe phoma stem canker in winter oilseed rape crops. Previous studies have agreed that in seasons experiencing elevated temperatures and increased rainfall, the efficacy of *R* genes is negatively influenced, and the stem canker severity is greater (Huang et al., 2006, 2018; Evans et al., 2008). Cotyledon assays showed clear differences between *R* genes in their resilience to maintain efficacy under elevated temperatures. Less is known about how these *R* genes respond individually to temperature in crops. Some work has been done in determining which months are most significant in affecting phoma severity; Huang et al. (2018) found phoma leaf spotting and stem canker severity to be linked to October and June average temperatures, respectively. This severity analysis did not explore the impact of maximum monthly temperatures. There is some evidence that maximum temperature may influence canker severity. A multiple linear regression analysis on 40 winter oilseed rape field experimental datasets by Evans et al. (2008) indicated that the mean maximum daily temperature and total rainfall (between 15 July and 26 September) produced the best prediction of the start date of the phoma leaf spotting epidemic, which is used to time the spraying of fungicides in autumn in the United Kingdom for all sites and growing seasons included. Maximum daily temperature and rainfall are important in stage one of the model described by Evans et al. (2008) relating to the date of leaf spotting in autumn. This study relates to stages two and three of the model (date of canker appearance in spring; severity of canker before harvest); for these stages, only temperature and host resistance are important. June is known to be a critical period in the development of the phoma stem canker; the most severe stage of the disease, the crown canker, occurs from May to July (West et al., 2001).

Conclusions drawn from investigations into the response of quantitative resistance at increased temperatures are somewhat conflicting. Huang et al. (2009) found, by analyzing stem cross-sections, the efficacy of quantitative resistance to be reduced when a cultivar with good quantitative resistance was exposed to an elevated temperature of 25°C compared to 15°C. While more severe cankers were observed on the cultivar with “little” quantitative resistance at 15°C, no significant difference between the two cultivars in stem canker severity at the higher temperature was observed, suggesting that temperature modifies the response of quantitative resistance to *L. maculans*. An experiment by Hubbard and Peng (2018) subjected *L. maculans*-inoculated *Brassica napus* cultivars with quantitative resistance to a temperature regime designed to mimic a heatwave, increasing to 32°C daytime temperature for 7 h before decreasing to 18°C for 7 h overnight. No difference in disease severity was found compared to plants grown at a moderate temperature regime of 22°C daytime/16°C overnight; suggesting that quantitative resistance can maintain efficacy at increased temperatures. It remains poorly understood how temperature affects the operation of quantitative resistance.

Here we aimed to determine how quantitative resistance and different *R* genes impacted the severity of the phoma stem canker of winter oilseed rape cultivars/breeding lines in field conditions, specifically in relation to the June maximum temperature. Second, we examined the effect of elevated temperature on the quantitative resistance response in stems during the colonization of stem tissues of the host *B. napus* by the pathogen *L. maculans* to develop stem canker.

## Materials and Methods

### Winter Oilseed Rape Field Experiments

A selection of *B. napus* breeding lines and cultivars with “good” or “little” quantitative resistance and major resistance (*R*) genes *Rlm4*, *Rlm7*, or *LepR3* were used in the field and controlled environment (CE) experiments. Field disease data and weather data were then analyzed to investigate the relationships between canker severity in different cultivars/breeding lines and maximum monthly temperatures throughout the growing seasons. The field experiments were run for three growing seasons (2016/17, 2017/18, and 2018/19) in England and France. There were two sites in 2016/17; Impington, Cambridgeshire, United Kingdom (lat. 52.253824°, long. 0.125801°) (the previous crop was wheat) and Châteauroux, France (lat. 46.5319°, long. 1.3758°) (the previous crop was wheat). There were two sites in 2017/18; Wisbech, Cambridgeshire, United Kingdom (lat. 52.695707°, long. 0.081937458) (the previous crop was pea) and Châteauroux, France (lat. 46.5319°, long. 1.3758°). However, the crop failed to establish at Châteauroux due to severe flea beetle damage. There were two sites in the United Kingdom in 2018/19: Callow, Herefordshire (lat. 51.994688°, long. −2.756194°) (the previous crop was wheat) and Wisbech, Cambridgeshire (lat. 52.619527°, long. 0.16128927°) (the previous crop was pea).

A total of 12 winter oilseed rape cultivars/breeding lines were selected for field experiments (Table 1). The rationale for the choice of genotypes was to include current cultivars/breeding lines with “good” or “little” quantitative resistance and *R* genes *Rlm4, Rlm7*, or *LepR3*. Current United Kingdom cultivars and breeding lines were included in the study to determine if temperature-resilient characteristics are present in commercially available oilseed rape cultivars. Seven of the cultivars/breeding lines have *R* genes with a “good” quantitative resistance background; DK Exception (*Rlm7*), Breeding line A (*Rlm7*), Adriana (*Rlm4*), Jet Neuf (*Rlm4*), Breeding line C (*Rlm4*), Breeding line E (*LepR3*), and Breeding line F (*LepR3*). Three of the cultivars/breeding lines have *R* genes and “little” quantitative resistance backgrounds; Breeding line B (*Rlm7*), Breeding line D (*Rlm4*), and Breeding line G (*LepR3*). Cultivar ES Astrid contains no known *R* genes but has a quantitative resistance background. Cultivar Incentive, which has no known *R* genes and “little” quantitative resistance, was used as a susceptible control. Breeding line C was not included in the first year as it was selected to replace a cultivar that did not establish in the first year of trials due to poor germination of the seed lot. The field experiments were arranged in randomized block designs with two or three replicates. Seeds were sown between late August and early September, at a density of 45 seeds/m^2^ in France and 55 seeds/m^2^ in the UK. Plots were 6 m^2^ for Châteauroux, France (2016/17, 2017/18), Impington (2016/17), and Wisbech, England (2017/18), and 8.6 m^2^ for Wisbech, England (2018/19) and Callow, England (2018/19).

**TABLE 1:** Winter oilseed rape cultivars and breeding lines tested in field experiments in 2016/17, 2017/18, and 2018/19 and a controlled environment (CE) experiment.

*R-*gene resistance	‘Good’ quantitative resistance	‘Little’ quantitative resistance
*Rlm7*	**Group 1** DK Exception^1^, Breeding line A^1^	**Group 2** Breeding line B^1^
*Rlm4*	**Group 3** Adriana^1^, Jet Neuf^1,2^, Breeding line C^1^*	**Group 4** Breeding line D^1,2^
*LepR3*	**Group 5** Breeding line E^1^, Breeding line F^1^	**Group 6** Breeding line G^1^
None	**Group 7** ES Astrid^1,2^	**Group 8** Incentive^1^, Breeding line H^2^

*There were 12 cultivars/breeding lines in the field experiment and 4 cultivars/breeding lines in the CE experiment (one breeding line is different; hence the 13 cultivars/breeding lines in the table).*

*Cultivars/breeding lines were categorized into eight groups, depending on their combination of R-gene and/or quantitative resistance.*

*Numbers in superscript refer to experiments in which the cultivar/breeding line was used; winter oilseed rape field experiments (1) and CE temperature-sensitivity assay (2).*

*Breeding lines A, B, C, D, E, F, G, and H are from NPZ and Jet Neuf is an NPZ cultivar. DK Exception is from DEKALB, Incentive is from DSV, Adriana is from Limagrain and ES Astrid is from Euralis.*

*Breeding line C was not included in the first year of field experiments.

### Frequency of Avirulent Alleles in *L. maculans* Populations

To determine the frequencies in the field experiment areas of virulent and avirulent alleles of *L. maculans* toward the *R* genes *Rlm4*, *Rlm7*, and *LepR3*, cultivar Drakkar with no *R* genes and no quantitative resistance was used for sampling *L. maculans* populations. Leaves of Drakkar with phoma leaf spot lesions were taken from Impington in autumn 2015 and Wisbech in autumn 2016 and 2017. *L. maculans* isolates from single pycnidia were obtained as described by Huang et al. (2018). Eight avirulent alleles of different effector genes in each *L. maculans* isolate were identified by inoculating the isolate onto cotyledons of a differential set of cultivars/lines carrying known *Rlm* genes (Huang et al., 2018).

### Phoma Stem Canker Severity Assessment on Different Cultivars and Breeding Lines

The severity of phoma stem canker was assessed in July, prior to harvest, and 15 plants were randomly pulled from each plot. The stems were cut at the base, immediately above the root collar and the area of necrotic tissue caused by phoma stem canker in the cross-section was scored using a scale from 0 to 6 (Pilet et al., 1998; Delourme et al., 2008); scale 0 = no affected tissue; scale 1 = 1–5% area affected; scale 2 = 6–25% area affected; scale 3 = 26–50% area affected; scale 4 = 51–75% area affected; scale 5 = 76–100% area affected, plant alive, and 6 = 100% area affected, stem broken or plant dead.

### Weather Data at Field Sites

The monthly average maximum temperature and total rainfall data were obtained for the five field site locations to assess their effects on phoma stem canker severity. Weather data were obtained from the NASA Langley Research Centre Atmospheric Science Data Centre Surface Meteorological and Solar Energy (SSE) web portal supported by the NASA LaRC POWER Project^1^. The average maximum monthly temperature for six months (from September to July) and maximum June temperature were used for analysis to investigate the effect of increased environmental temperatures.

### Effects of Temperature on the Growth Rate of Different *L. maculans* Isolates

The growth of *L. maculans* isolates *in vitro* was assessed at both 20°C and 25°C to ensure any differences in phenotype were not caused by differences in pathogen growth rate. The growth rates of *L. maculans* isolates v23.1.3 (*Av1-4-5-6-7*; avirulent against *Rlm4*) and v23.11.9 (*Av1-5-6-7*; virulent against *Rlm4*) were compared at 20 and 25°C; both isolates were derived from a single cross (Balesdent et al., 2001). Mycelial disk inoculum was placed in the center of 9 cm diameter Petri dishes of V8 agar amended with penicillin (20 mg L^–1^ filter sterilized) and streptomycin (40 mg L^–1^ filter sterilized). These were then stored for 24 h in darkness at 20°C before transfer to the CE chamber for further growth. Six replicate Petri dishes were prepared per treatment. Photographs were taken daily over a 5-day period using a NEX-5R camera (Sony) with a 40.4–49 mm lens. Photos were taken from a fixed height and under controlled lighting to reduce image distortion and give color consistency between treatments. Image J software was used to trace the circumference of the isolated colony in each image using the freehand tool. This method was used to provide more accurate results than measuring colony radius with a ruler as isolates of *L. maculans* often grow with an irregular margin rather than a perfect circular perimeter. The dark orange V8 agar provided a clear contrast to the white mycelia allowing the areas of fungal growth to be clearly identified.

### Plant Growth and Stem Inoculation for Controlled Environment Assay

The effect of temperature on the quantitative disease resistance and the role of *R* genes during the second symptomless stage of colonization was investigated by the inoculation of the stem bases of *B. napus* young plants with *L. maculans* isolates. Cultivars/breeding lines possessing four different combinations of resistance genotypes were selected; susceptible background with no *R* genes (Breeding line H); “good” quantitative resistance background with no known *R* genes (ES Astrid); susceptible background with *Rlm4* (Breeding line D), and “good” quantitative resistance background with *Rlm4* (Jet Neuf) (Table 1). Two isolates of *L. maculans* were used for inoculation; one avirulent and the other virulent against *Rlm4*; v23.1.3 (*AvrLm4*), and v23.11.9 (*avrLm4*), respectively. Plants were treated with the virulent *L. maculans* isolate to remove any resistance response caused by major *R* gene interactions. Thus, any differences in resistance response observed in these plants were due to differences in the quantitative resistance background. For each of the four treatments (inoculation with avirulent isolate at 20°C, inoculation with avirulent isolate at 25°C, inoculation with virulent isolate at 20°C, and inoculation with virulent isolate at 25°C), 15 plants of each cultivar/breeding line were subdivided into three sub-blocks of five, which were arranged randomly between four trays each containing 15 plants (three sub-blocks). Plants were grown in a 1:1 ratio of MiracleGro and John Innes No 3 compost, in 6 cm × 6 cm wide and 8 cm deep pots, inside CE cabinets at a constant temperature of 20°C (12-h light/12-h dark). Light intensity at plant height was measured to be 320 μmol/m^2^/s. Plants were divided into two groups 24 h prior to inoculation; half were transferred to 25°C, the rest remaining at 20°C. Plants were inoculated after 6-weeks of growth in the CE cabinets by placing a 1 cm^2^ square piece of sponge cloth soaked in 10^7^ ml^–1^ conidial suspension over a 1 cm cut in the stem, then wrapping with Parafilm to secure it in place.

### Image-Based Canker Severity Assessment and Measurement of Plant Health

**FIGURE 1:**
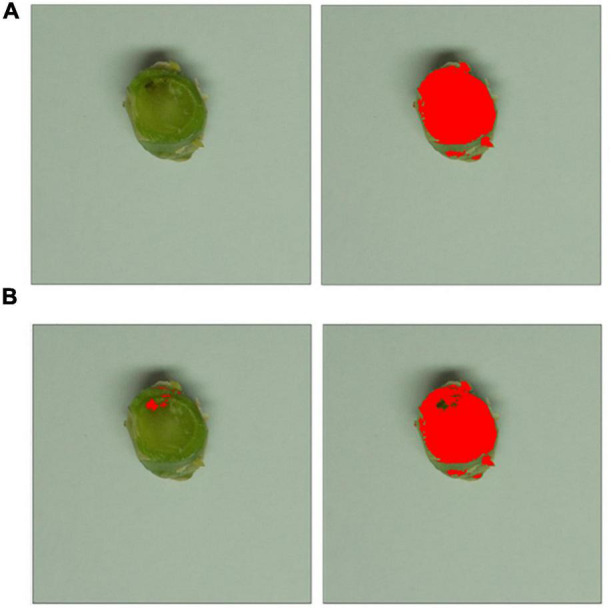
Image-based canker severity assessment workflow. Photos of stem pieces for each treatment were cropped to isolate each of the individual stems to allow more in-depth statistical analysis. **(A)** The saturation threshold level was adjusted for each individual image to ensure the cross-sectional area was totally masked, as shown in red on the right-hand side. **(B)** Brightness and hue threshold filters were applied, identifying necrotic tissue (shown in red on the left) and healthy green tissue (shown in red on the right).

Assessment of plant health and canker severity was done at 6-weeks post-inoculation. To assess plant health, the following measurements were taken for each plant; leaf number, plant height (stem base to the tip of the longest leaf), and stem thickness (measured with a digital caliper). To assess stem canker severity, 1 cm long pieces of the stem were cut at 1 cm below the inoculation site and photographed as previously described. Photos were then batch-cropped into fifteen photos of stem pieces, each measuring 815 pixels × 815 pixels, to improve accuracy before statistical analysis. These were then analyzed with Image J (Schneider et al., 2012) to determine the percentage area of the necrotic tissue discolored by the disease to assess the severity of the stem canker (Figure 1). Saturation was adjusted for each image to ensure the cross-section of the stem was fully covered in the analysis. Healthy tissue was identified through setting the color threshold parameters, in HSB (hue, saturation, brightness) mode to brightness min 82, hue min 42. Settings for necrotic discolored tissue were brightness max 81 and hue max 41. The Analyze Measure function was then used to measure the pixels in the filtered areas.

### Statistical Analysis

The statistical analyses of the data were all done using GenStat statistical software (VSN International, 2020). ANOVA was done to test the effects of cultivars/breeding lines with *R* gene resistance, “good” quantitative resistance, *R* gene resistance with “good” quantitative resistance, or susceptible background on stem canker severity score. ANOVA was also done to test the effects of oilseed rape cultivar/breeding line and temperature on plant height, leaf number, and stem diameter of the oilseed rape cultivars and breeding lines tested. The *post hoc* test with Fisher’s least significant difference (LSD) calculated at *P* = 0.05 was used to separate the difference between means of treatments. Correlation analysis for canker severity score against mean monthly maximum temperature was done to identify the month with the greatest temperature effect on phoma stem canker severity score. Then, the relationship between the stem canker severity score and the highest maximum temperature recorded in June was analyzed using linear regression. Differences between cultivars/breeding lines were tested using comparative analysis of position and parallelism of linear regression (i.e., linear regression with groups).

## Results

### Frequency of Avirulent Alleles in *L. maculans* Isolates

**FIGURE 2:**
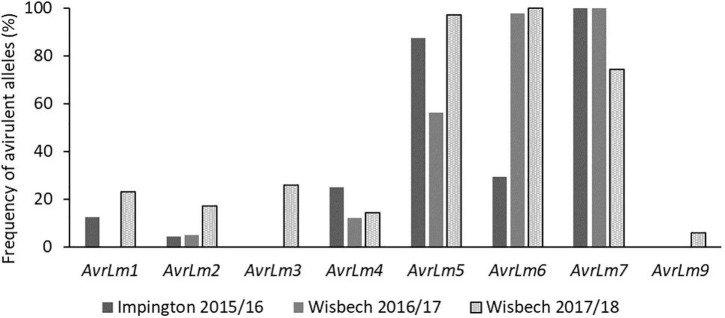
Frequency of avirulent alleles in *Leptosphaeria maculans* isolates taken from field locations in Wisbech and Impington, United Kingdom. To determine the frequencies of virulent/avirulent alleles of *L. maculans* present in the field experiment areas toward the *R* genes *Rlm4*, *Rlm7*, and *LepR3*, cultivar Drakkar with no *R* genes and no quantitative resistance was used for sampling *L. maculans* populations. Leaves of Drakkar with phoma leaf spot lesions were taken from Impington in autumn 2015 and Wisbech in autumn 2016 and 2017 for obtaining *L. maculans* isolates. Avirulent alleles of different effector genes (*Avr*) genes in each *L. maculans* isolate were identified by inoculating the isolate onto cotyledons of a differential set of cultivars/breeding lines carrying known *Rlm* genes (Huang et al., 2018).

The proportions of the avirulent alleles of *AvrLm1, AvrLm2, AvrLm3, AvrLm4, AvrLm5, AvrLm6, AvrLm7*, and *AvrLm9* were assessed in *L. maculans* isolates obtained from leaf lesions taken from experimental locations in Impington (autumn 2015) and Wisbech (autumn 2016 and 2017). The frequency of isolates with *AvrLm7* in Wisbech decreased in 2017/18 (74.3%) compared to 2015/16 and 2016/17 (100%) (Figure 2) and would be expected to decrease further during 2018/19. Thus, cultivars/breeding lines with *Rlm7* would be expected to have good resistance against phoma stem canker in the first year; that would deteriorate during the second and third year of the field experiments as the *avrLm7* races increased in frequency. Most of the isolates tested were found to have the virulent alleles *avrLm1* (87.5% for Impington 2015/16, 100% for Wisbech 2016/17, and 77.1% for Wisbech 2017/18) and *avrLm4* (75% for Impington 2015/16, 87.8% for Wisbech 2016/17, and 85.7% for Wisbech 2017/18) which confer virulence to resistance genes *Rlm1* or *Rlm4*, respectively. Since the effector gene *AvrLm1* is recognized by the resistance genes *Rlm1* and *LepR3*, cultivars/breeding lines containing *LepR3* would be expected to show severe phoma stem canker at these sites. Similarly, cultivars/breeding lines containing *Rlm4* would also be expected to be susceptible as the effector gene *AvrLm4-7* is recognized by the resistance gene *Rlm4* (Pilet et al., 1998).

### Phoma Stem Canker Severity on Different Cultivars and Breeding Lines

**FIGURE 3:**
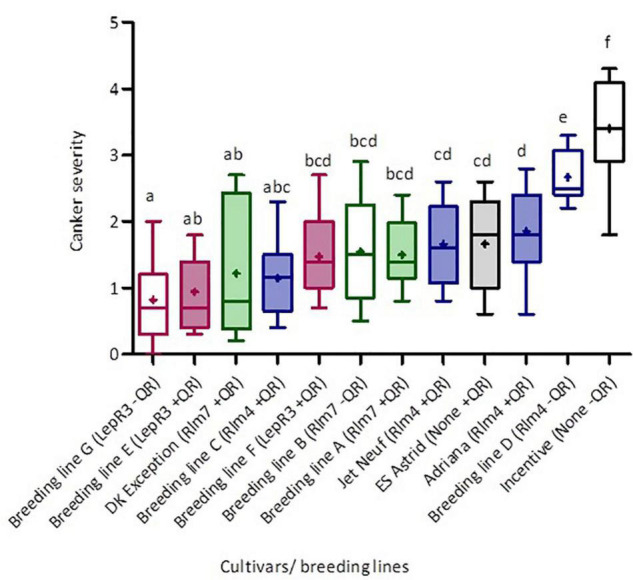
Distribution of phoma canker severity scores for winter oilseed rape cultivars/breeding lines with different *R* genes and/or quantitative resistance. Cultivars/breeding lines were grown at five locations over three growing seasons (Chateauroux, France, 2016/17; Impington, United Kingdom, 2016/17; Wisbech, United Kingdom, 2017/18 and 2018/19; and Callow, Herefordshire, United Kingdom, 2018/19). Each box-plot shows the mean (cross) and median (line) scores for each cultivar/breeding line. Upper and lower box boundaries denote the 25th and 75th percentiles and whiskers indicate the minimum and maximum severity scores. Basal stem canker severity (Scale 0–6; Lô-Pelzer et al., 2009) was scored on fifteen plant stems randomly sampled from each plot. Colors represent the different *R* genes in cultivars/breeding lines; pink is *LepR3*, green *Rlm7*, blue *Rlm4*, and black no known *R* gene. Shaded boxes denote cultivars with higher levels of quantitative resistance as indicated by breeders. Average scores sharing the same letter are not statistically different (*P* < 0.05) in multiple comparisons using Fisher’s least significant difference (LSD) test.

The phoma stem canker severity scores for the twelve winter oilseed rape breeding lines/cultivars with different combinations of *R* genes and/or quantitative resistance included in the field experiments in England and France were analyzed. Figure 3 shows the distribution of phoma canker severity scores between cultivars/breeding lines. The cultivar Incentive (“little” quantitative resistance, no known *R* genes) had the greatest canker severity (mean severity score = 3.88), two times greater than that of the cultivar ES Astrid (with quantitative resistance only). Breeding line G (“little” quantitative resistance and *LepR3*) had the smallest average severity score (0.82). The greatest variance in stem canker severity was observed in cultivar DK Exception (“good” quantitative resistance and *Rlm7*) and the smallest in Breeding line D (“little” quantitative resistance and *Rlm4*).

Cultivars/breeding lines with the same *R* gene and “good” or “little” categorization of quantitative resistance were grouped together to allow cross-comparison (Table 2), and the material containing the same *R* genes is color-coded in Figure 3. Fisher’s least significant comparison test was done to test for significant differences between *R* genes and quantitative resistance in average stem canker severity. Large differences were seen in *R* gene effects in cultivars/breeding lines with “little” quantitative resistance; cultivars/breeding lines with *Rlm7*, *Rlm4*, *LepR3*, or no known *R* gene were all significantly different from each other. However, in cultivars/breeding lines with quantitative resistance, there were no *R* gene effects. No significant differences were found for *Rlm7* and *LepR3* cultivars/breeding lines between those with “good” and “little” quantitative resistance. However, for cultivars/breeding lines with *Rlm4* and cultivars/breeding lines with no known *R* gene, significant differences were found between those with “good” and “little” quantitative resistance. In both cases, those with “good” quantitative resistance had a significantly smaller score. Quantitative resistance had the largest protective effect against stem canker caused by *L. maculans* in the absence of *R* genes.

**TABLE 2:** Fisher’s least significance comparison of average canker severity scores for 12 winter oilseed rape cultivars/breeding lines grouped by single *R* gene and quantitative resistance.

*R* gene	Quantitative resistance*		*R* gene mean
	“Little”	“Good”	
*Rlm7*	1.53b	1.36b	**1.421**
*Rlm4*	2.66c	1.57b	**1.830**
*LepR3*	0.82a	1.20ab	**1.072**
None	3.39d	1.66b	**2.520**
**Quantitative resistance mean**	**2.074**	**1.433**	

*Average scores sharing the same letter were not statistically different at P < 0.05 in multiple comparisons with Fisher’s least significant difference (LSD) test. Values in bold are overall means for genotypes with R gene-mediated or quantitative resistance.

### Effect of June Temperature on Canker Severity in Cultivars and Breeding Lines Varying in *R* Gene-Mediated and/or Quantitative Resistance

**FIGURE 4:**
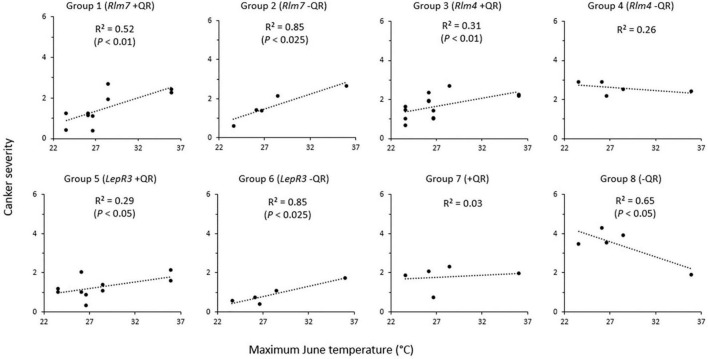
Relationship between phoma stem canker severity of winter oilseed rape cultivars/breeding lines and maximum June temperature. Twelve cultivars/breeding lines (see Table 1) were grown in 2–3 replicate blocks over three growing seasons at five locations in Chateauroux, France (2016/17); Impington, Cambridgeshire, United Kingdom (2016/17); Wisbech, Cambridgeshire, United Kingdom (2017/18 and 2018/19); and Callow, Herefordshire, United Kingdom (2018/19). Basal stem canker severity (Scale 0–6; Lô-Pelzer et al., 2009) was scored on 15 plant stems randomly sampled from each plot.

An initial correlation analysis for the canker severity score and mean monthly maximum temperature was done to identify the month with the greatest temperature effect on phoma stem canker severity score. June was found to have the greatest influence, with a correlation coefficient of *r* = 0.33 (Supplementary Table 1). The regression analysis of the relationship between the greatest recorded June temperature and phoma stem canker severity score in cropping years 2016/17, 2018/19, and 2018/19 showed that there were differences between genotypes (Figure 4). The highest maximum June temperature was 35.97°C in Chateauroux, France (June 22, 2017) and the lowest maximum June temperature was 23.59°C in Wisbech (June 25, 2018).

Groups 1 and 2 (cultivars/breeding lines with *Rlm7* and with “good” or “little” quantitative resistance) both had a positive correlation between the maximum June temperature and phoma stem canker severity. This correlation was stronger in the cultivars/breeding lines with “little” quantitative resistance (*R*^2^ = 0.85, *P* < 0.025) than that for the cultivars/breeding lines with “good” quantitative resistance (*R*^2^ = 0.52, *P* < 0.01). Groups 3 and 4 (cultivars/breeding lines with *Rlm4*, and “good” or “little” quantitative resistance) showed a much weaker relationship of phoma stem canker severity with maximum June temperature (*R*^2^ = 0.31, *P* < 0.01 and *R*^2^ = 0.26, *P* > 0.05 respectively). With quantitative resistance, a positive correlation was observed, but with “little” quantitative resistance (Breeding line D), there was no significant correlation. Group 5 (*LepR3* with “good” quantitative resistance) (Breeding lines E and F) and group 6 (*LepR3* and “little” quantitative resistance) (Breeding line G) followed a similar trend to groups 1 and 2; both showed a positive correlation between phoma stem canker score and maximum June temperature, with a stronger correlation in cultivars/breeding lines with “little” quantitative resistance (*R*^2^ = 0.86, *P* < 0.025) than those with “good” quantitative resistance (*R*^2^ = 0.29, *P* < 0.05). Group 7 (cultivar ES Astrid with “good” quantitative resistance and no known *R* genes) showed no significant correlation between the canker severity score and maximum June temperature (*R*^2^ = 0.034). Group 8 (cultivar Incentive with no known *R* genes and “little” quantitative resistance) showed a negative correlation (*R*^2^ = 0.65, *P* < 0.05) with reduced phoma stem canker severity at the higher temperatures. Analysis of position and parallelism based on cultivar/breeding line *R* genes compared groups with *R* genes against susceptible cultivar Incentive. Cultivars/breeding lines with *Rlm7* (*P* < 0.01) and *LepR3* (*P* < 0.05) both had significantly different slopes, but those cultivars/breeding lines with *Rlm4* did not (*P* = 0.061). The intercept was found to be significantly different from Incentive for all cultivars/breeding lines; *Rlm7* (*P* < 0.001), *Rlm4* (*P* < 0.05), and *LepR3* (*P* < 0.01).

### Effects of Temperature on the Growth Rate of *L. maculans* Isolates in Culture

**FIGURE 5:**
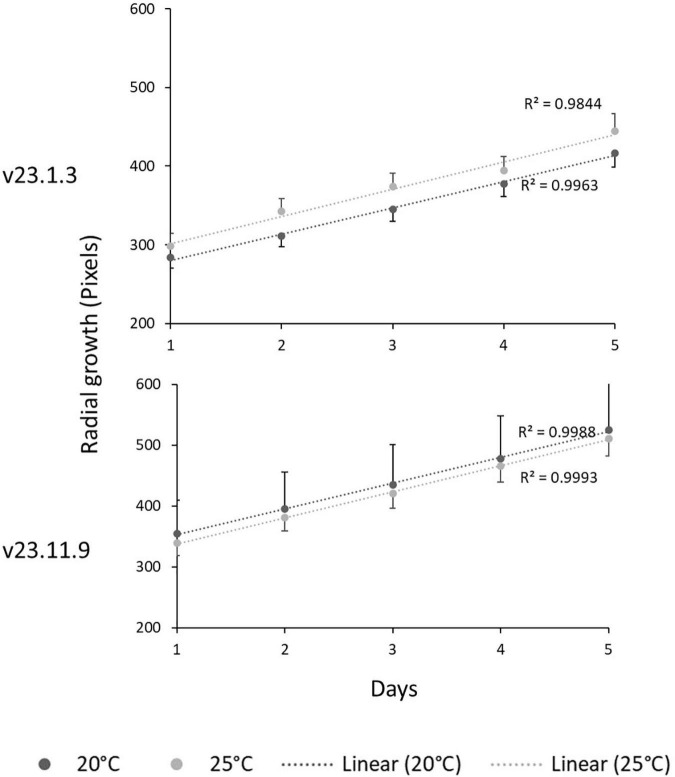
Effects of temperature on the radial growth rates of *L. maculans* isolates v23.1.3 and v23.11.9 at 20 and 25°C. Mycelial disks were transferred from fungal colonies onto V8 media Petri dishes incubated at a constant temperature of 20 or 25°C in darkness. Photographs were taken daily at regular time points for 5 days. The area of fungal growth was analyzed using Image J. An ANOVA showed that temperature had not significantly affected radial growth rate for v23.1.3 (*P* = 0.322) or v23.11.9 (*P* = 0.971). Error bars indicate the standard error of the mean (5 df).

The two *L. maculans* isolates used in this study, v23.1.3 and v23.11.9, were grown at 20 and 25°C under CE conditions and measured every 24 h for 5 days to determine the effect of temperature on their growth (Figure 5). The growth rates of both isolates were not affected by temperature. The perimeters of the v23.1.3 cultures were slightly larger at 25°C than at 20°C, whereas the perimeters of the v23.11.9 cultures were marginally larger at 20°C. However, neither of these differences were significant.

### Effects of Temperature on Phoma Stem Canker Severity in *B. napus* With Quantitative and/or *R* Gene-Mediated Resistance Under Controlled Environment Conditions

**FIGURE 6:**
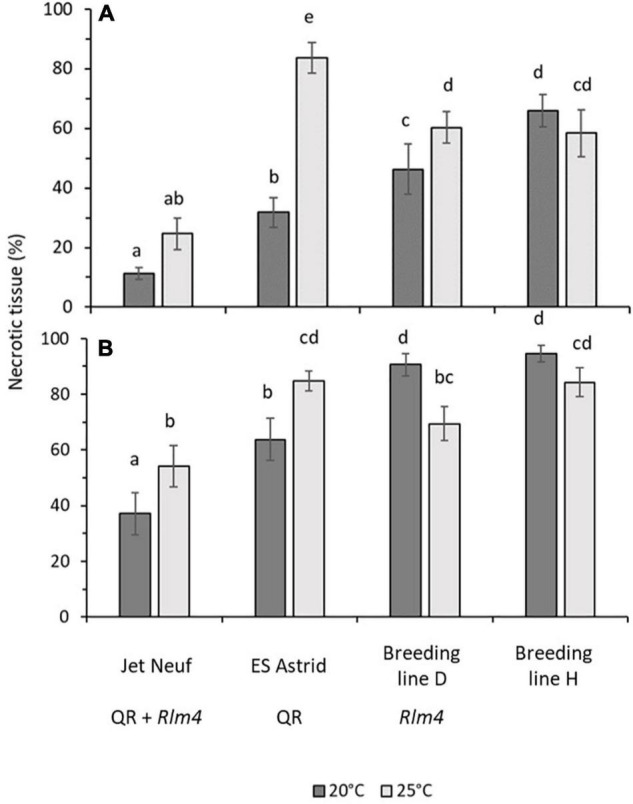
Canker severity in four winter oilseed rape cultivars/breeding lines at 20 or 25°C. Stems of 6-week-old plants were inoculated with *L. maculans* isolates v23.1.3 (*AvrLm4*) **(A)** or v23.11.9 (*avrLm4*) **(B)** by wrapping a sponge soaked in 10^7^ mL^– 1^ conidial suspension over a 1 cm cut in the stem with Parafilm. Pieces 1 cm long of the stem were cut 1 cm above the inoculation site and photographed at 6 weeks post-inoculation. The mean percentage area of necrotic tissue in stem pieces was calculated from the area of tissue discolored by the disease and the total area analyzed using ImageJ. A total of 15 plants per treatment for each cultivar/breeding line were assessed. Error bars indicate the standard error of the mean (14 df). Average scores sharing the same letter are not statistically different (*P* < 0.05) in multiple comparisons using Fisher’s LSD test.

The effect of increased temperature, from 20 to 25°C, on canker severity for four winter oilseed rape cultivars/breeding lines with different resistance profiles was determined, 6 weeks following inoculation with *L. maculans* isolates avirulent (v23.1.3) or virulent (v23.11.9) against *Rlm4* (Figure 6). *Rlm4* was chosen as the *R* gene in this study as significant differences between cultivars/breeding lines with “little” and “good” quantitative resistance had previously been observed in the field experiment (Table 2). As expected, canker severity was greater when the different plant genotypes were inoculated with the virulent (Figure 6B) rather than the avirulent isolate (Figure 6A). Cultivar Jet Neuf (“good” quantitative resistance and *Rlm4*) showed the smallest canker severity at both temperatures when inoculated with the avirulent rather than the virulent isolate. No significant difference was seen between the two temperatures for the inoculation with the avirulent *L. maculans* isolate v23.1.3 (11.3 and 24.6% necrosis for 20 and 25°C, respectively); however, the virulent isolate produced significantly greater amounts of necrosis at 25°C (54.1%) compared to 20°C (37.1%). Cultivar ES Astrid (“good” quantitative resistance, no known *R* genes) performed well against isolate v23.1.3 at 20°C with an average necrotic area of 31.8%. However, this cultivar resistance lost efficacy at 25°C, with over twice as much necrotic tissue area (83.7%). When inoculated with the virulent isolate v23.11.9, a significant difference was also seen between the temperatures (63.7 and 84.8% necrotic tissue for 20 and 25°C, respectively). Breeding line D (“little” quantitative resistance and *Rlm4*) also showed a significant difference between the two temperatures when inoculated with the avirulent v23.1.3 isolate (46.3 and 60.3% necrotic tissue area at 20 and 25°C, respectively). When inoculated with the virulent isolate, this temperature effect was reversed, with a statistically significantly greater necrotic tissue area (90.7%) found at 20°C, compared to 69.4% at 25°C. Breeding line H (“little” quantitative resistance, no known *R* genes) did not exhibit any significant temperature effect, although for both isolates the canker severity was slightly less at the higher temperature. When inoculated with the isolate v23.1.3, the necrotic tissue area was 66 and 58.4% at 20 and 25°C, respectively. When inoculated with the isolate v23.11.9, it was 94.7 and 83.3% at 20 and 25°C, respectively. Both differences were not significant.

### Effects of Temperature on Plant Growth Parameters of the Oilseed Rape Cultivars and Breeding Lines Tested

Plant health assessments showed very little difference between the temperatures of 20°C and 25°C (Table 3). Although some differences were seen in stem diameter, these were not significant except for cultivar ES Astrid with a larger stem diameter at 20°C. However, this difference did not affect the image analysis of stem canker because percentages of the area that were necrotic were determined. Cultivar ES Astrid grew better at 20°C than at 25°C; plants grew taller and had more leaves, but these differences were not significant. Breeding line D grew taller at 25°C than 20°C, significantly when inoculated with isolate v23.11.9; however, significantly more leaves were produced at 20°C when inoculated with this isolate. Breeding line H grew significantly taller at 25°C than at 20°C when inoculated with isolate v23.11.9.

**TABLE 3:** Effect of increased temperature from 20 to 25°C on average plant height, leaf number, and total stem diameter of four winter oilseed rape cultivars/breeding lines, inoculated with v23.1.3 or v23.11.9 isolates of *Leptosphaeria maculans*.

Isolate		Jet Neuf		ES Astrid		Breeding line D		Breeding line H	
		20°C	25°C	20°C	25°C	20°C	25°C	20°C	25°C
v23.1.3	Height (cm)	30.4	29.3	26.3	25.9	33.4	35.9	27.1	27.9
	Leaf number	6.3	6.4	4.9	4.8	5.9	5.6	7.4	6.9
	Stem diameter (mm)	4.2	4.4	4.3*	3.6	4.6	4.9	5.4	5.3
v23.11.9	Height (cm)	27.9	28.3	26.5	25.5	30.2*	34.6	23.1*	29.8
	Leaf number	5.3	5.7	5.1	4.8	6.0*	5.3	7.9	7.6
	Stem diameter (mm)	4.3	3.8	4.1	4.2	4.7	5.0	4.4	4.9

To compare the differences between variables for v23.1.3, use least significant differences (at P < 0.05) for between heights = 2.014; for between leaf numbers = 0.576 and for between stem diameters = 0.467. To compare the differences between variables for v23.11.9, use least significant differences (P < 0.05) for between heights = 2.558; for between leaf numbers = 0.632 and for between stem diameters = 0.598. *Significant at P < 0.05.

## Discussion

### Weather Influences Phoma Stem Canker Severity in Genotypes With Different *R* Genes and/or QR

This study confirmed the findings of previous work on the effect of temperature on phoma stem canker severity. While the average June temperature showed a correlation with phoma stem canker severity, a stronger positive relationship was observed for the maximum June temperature recorded and phoma stem canker severity score. This new observation supports the suggestion that more cultivars will require temperature-resilience to perform successfully in years experiencing high June temperatures, as these are predicted to increase with climate change (Evans et al., 2008; Pullens et al., 2019).

Cultivars/breeding lines with the *R* genes *Rlm7*, *Rlm4*, and *LepR3* responded differently in terms of phoma canker severity to the maximum June temperature. Cultivars/breeding lines with *Rlm7* showed a positive correlation with canker severity increasing with temperature. An alternative explanation could be that *L. maculans* isolates differed between the sites that were tested. Virulent isolates containing *avrLm7* alleles were present in France; the field site Châteauroux experienced the highest maximum June temperature. It is therefore possible that *L. maculans* races, rather than a temperature-sensitive *Rlm7* affected phoma stem canker severity. A recent study of the *Avr* frequencies present in 30 *L. maculans* isolates sampled in Le Rheu, France found 63% had virulence against *Rlm7* (Bousset et al., 2020). Cultivars/breeding lines with *LepR3* had the smallest average canker score, but it also showed a positive relationship with the maximum June temperature.

Breeding line D (“little” quantitative resistance and *Rlm4*) had the second-largest average canker severity; this genotype showed an insignificant correlation between maximum June temperature and canker severity. This could be due to *Rlm4* not being a temperature-sensitive *R* gene; alternatively, a significant proportion of *L. maculans* isolates with virulence against *Rlm4* at the experiment sites, as shown in Figure 2, may have an effect. Analysis of *L*. *maculans* populations from 13 sites, 11 of which were in the United Kingdom, by Huang et al. (2018) found mean frequencies of *AvrLm4* to be 41%, less than that of *AvrLm7* which was 100%. Within the 30 *L. maculans* isolates sampled in Le Rheu, France, 100% were found to show virulence against *Rlm4* (Bousset et al., 2020). This suggests that *Rlm4* gene-mediated resistance would be rendered at least partially ineffective, explaining the greater canker severity observed in Breeding line D. However, genotypes with *Rlm4* and quantitative resistance had more severe stem canker severity scores at a higher temperature, suggesting that races were “not the end of this story”. Collectively, this suggests that more research is needed on the sampling of isolates from experimental fields together with the characterization of their *Avr* gene profiles.

### Lower Temperatures Are More Conducive to Canker Development for Susceptible Cultivars and Breeding Lines

A significant negative correlation of phoma canker severity in field experiments with maximum June temperature was seen for cultivar Incentive, which lacks both known *R* genes and quantitative resistance (Figure 4). Susceptible Breeding line H plants also had a greater amount of necrosis in the stem at 20°C compared to 25°C in the CE experiment, although this difference was not significant when tested separately for each *L. maculans* isolate tested. Together, it may be a result of a greater temperature optimum for PTI. Increased temperatures (23–32°C) have been reported to enhance PAMP signaling in *Arabidopsis thaliana*; on the contrary, ETI has a lower temperature optimum of 10–23°C (Cheng et al., 2013).

### *R* Genes Operate in the Stems of Young Plants Under Controlled Environment Conditions

Results from the field experiments suggested that *R* genes are operating alongside quantitative resistance in June to influence the phoma stem canker severity. Through inoculating stems of young plants, any resistance brought about by *R* genes operating in the leaves was circumvented in stems in the CE experiment. As a control, axenic growth of *L. maculans* was monitored at 20 and 25°C, but no difference in radial growth rate was observed (Figure 5), which is not inconsistent with previous publications (Newbery et al., 2020). While the subtle environmental changes like temperature may influence molecular processes in organisms, it is also rational to assume that organisms can compensate for such changes at least over a certain range. The similar growth rates of *L. maculans* at 20 and 25°C reflect this dynamic range and fit with naturally occurring temperatures in June. Data in Table 3 demonstrated that any observed difference in symptom development under both temperature regimes did not result from different growth rates. Furthermore, it has previously been shown that *L. maculans* can cause disease at both 20 and 25°C (Huang et al., 2006).

The *L. maculans* isolate avirulent to *Rlm4* was found to cause significantly less necrotic tissue in stems of a cultivar with *Rlm4* grown at 20°C than that grown at 25°C. This suggests that *Rlm4* has a protective or suppressive effect against the pathogen growth in the stems of young plants. Previous work showed that *R* genes operate in the leaves of young plants during the autumn to prevent leaf spotting (Rimmer and van den Berg, 1992; Fitt et al., 2006). To confirm this hypothesis, more stem inoculation experiments should be done using near-isogenic lines with or without individual *R* genes. However, little work has been done on the operation of *R* genes in stems. There is a need to test more cultivars with different *R* genes using stem inoculation, ideally to test near-isogenic lines with or without single *R* genes.

### Quantitative Resistance May Protect *R* Gene-Mediated Resistance at High Temperatures

Results of field experiments suggested that quantitative resistance may act to reduce the effect of increasing maximum June temperature on the phoma stem canker severity when combined with *R* genes. This correlation between the maximum June temperature and phoma stem canker severity was weaker for *Rlm7* and *LepR3* cultivars/breeding lines with quantitative resistance compared to those with “little” quantitative resistance. When quantitative resistance was present in a cultivar/breeding line (e.g., ES Astrid) with no known *R* genes, no relationship with maximum June temperature was seen. These findings suggested that quantitative resistance shows temperature-resilience in crops and can buffer a plant resistance response against high temperature, maintaining the efficacy of the plant resistance. However, in the CE stem inoculation experiments, cultivars ES Astrid (with “good” quantitative resistance) and Jet Neuf (*Rlm4* with “good” quantitative resistance) were both found to have a significantly smaller amount of necrotic tissue at 20°C than at 25°C. This suggests that, under a sustained temperature of 25°C, the efficacy of quantitative resistance is reduced. This finding is consistent with previous publications on the temperature sensitivity of quantitative resistance under CE conditions (Huang et al., 2009). One possible explanation for the difference in the performance of cultivar ES Astrid between the field and CE experiments could be the period in which the plant is exposed to elevated temperatures. This finding is supported by CE experiments that mimicked heat waves occurring in Canadian Prairies; gradual increases in temperature from a 7-h night-time period at 18°C to reach a 7-h daytime of 32°C did not change quantitative resistance against *L. maculans*, suggesting that quantitative resistance maintains its efficacy when the increased temperature is not sustained for a long period (Hubbard and Peng, 2018). While quantitative resistance appears to provide a mechanism to reduce the effect of elevated temperature, this may be rendered ineffective if this higher temperature is sustained over a long period.

Although cultivars/breeding lines were classified as having quantitative resistance or not, the resistance mechanisms of different cultivars/breeding lines with quantitative resistance may be completely different. Furthermore, other differences in the genetic backgrounds of these cultivars/breeding lines may also influence their response to the environment and impact the severity of phoma stem canker. Thus, there are clear limitations to this study. Nevertheless, in the absence of a set of oilseed rape lines differing only in their quantitative resistance trait loci, this set of genotypes provides a good choice for investigating the effect of temperature on the quantitative resistance response.

It is not known if increased levels of quantitative resistance are linked with a reduction in fitness. A review by Brown (2002) on yield penalties of disease resistance in crops suggested that plants with good quantitative resistance could suffer from a fitness penalty. The evidence behind this proposal came from the observations of Vanderplank (1984) that quantitative resistance can be lost due to masking by single *R* genes or if not exposed to the pathogen. Quantitative resistance genes could be linked to genes involved in yield, resulting in linkage drag if these resistance genes were to be introgressed. More research is needed in this area to fully understand any potential trade-offs in important traits, such as yield, that may be linked to greater levels of quantitative resistance in oilseed against phoma stem canker.

## Conclusion

Results from field experiments suggest that temperature-resilient quantitative resistance is currently available in some oilseed cultivars. For example, ES Astrid (“good” quantitative resistance, no known *R* genes) showed no significant correlation between canker severity score and maximum June temperature. However, ES Astrid had significantly smaller amounts of necrotic tissue at 20°C than at 25°C when inoculated with both virulent and avirulent *L. maculans* isolates under CE conditions. We suggest that the efficacy of quantitative resistance is maintained at increased temperature but not when these elevated temperatures are sustained for long periods of time under CE conditions.

The effectiveness of *Rlm4* mediated resistance in the stem also appears to be reduced when plants are subjected to a prolonged elevated temperature of 25°C. Significantly more necrotic tissue was found at 25°C than 20°C after Breeding line D (“little” quantitative resistance and *Rlm4*) was inoculated with an avirulent *L. maculans* isolate. The reverse was seen when the same line was inoculated with a virulent *L. maculans* isolate. However, in Jet Neuf (“good” quantitative resistance and *Rlm4*) there was no significant difference in the amount of necrotic tissue between the two temperatures, when inoculated with an avirulent isolate. Therefore, in years experiencing warmer summers, as have been predicted to result from climate change in the United Kingdom, a combination of temperature-resilient *R* genes and a good quantitative resistance background will be required to protect oilseed crops from phoma stem canker.

Furthermore, the results of the CE experiments show that both quantitative resistance and *R* gene resistance operate in the stem by either preventing or suppressing the growth of *L. maculans*, subsequently reducing stem canker severity. This is important to growers as yields can be significantly reduced by phoma stem canker developing in the summer months. There may be scope to reduce this damage in the future by assessing new cultivars to determine their level of stem resistance and ability to maintain resistance at elevated temperature, using stem base inoculation methods in CE assays. However, it would be advised that the temperatures in these assays would be set to simulate the types of heatwaves forecast to become more common as global warming advances. Temperatures should be set to fall at night rather than maintain a constant temperature throughout.

From this study, it could be suggested that *Rlm4* is a weaker, yet more temperature-resilient, *R* gene compared to *Rlm7* and *LepR3*. For cultivars/breeding lines with *Rlm4*, significant differences were found between those with “good” and “little” quantitative resistance. However, it must be remembered that high frequencies of virulent isolates with *avrLm4* alleles were found in two of the field experiment locations. Cultivars/breeding lines with *Rlm4*, with “good” or “little” quantitative resistance, showed a much weaker relationship of phoma stem canker severity with maximum June temperature compared to cultivars and breeding lines with *Rlm7* and *LepR3*.

## Data Availability Statement

The original contributions presented in the study are included in the article/Supplementary Material, further inquiries can be directed to the corresponding author.

## Author Contributions

YH, HUS, SR, CP, LG, and KN: experimental design. AQ and KN: statistical analysis. KN and LG: lab work. KN and CP: field work. BDLF and HUS: project administration. HUS: supervision. KN and HUS: writing – original draft. KN, HUS, YH, AQ, and BDLF: writing – review and editing. All authors contributed to the article and approved the submitted version.

## Conflict of Interest

KN and CP are employed by LS Plant Breeding Ltd. SR is employed by NPZ Innovation GmbH. The authors declare that this study received funding from LS Plant Breeding. The funder had the following involvement in the study: Contribution of breeding lines and field experiments. The funder was not involved in the study design, collection, analysis, interpretation of data, the writing of this article or the decision to submit it for publication. The remaining authors declare that the research was conducted in the absence of any commercial or financial relationships that could be construed as a potential conflict of interest.

## Publisher’s Note

All claims expressed in this article are solely those of the authors and do not necessarily represent those of their affiliated organizations, or those of the publisher, the editors and the reviewers. Any product that may be evaluated in this article, or claim that may be made by its manufacturer, is not guaranteed or endorsed by the publisher.

## Footnotes

1
https://power.larc.nasa.gov/data-access-viewer/
