# Supplementary material for: Influence of Elevated Temperatures on Resistance Against Phoma Stem Canker in Oilseed Rape
Source: Front Plant Sci. 2022 Mar 2;13:785804. doi: 10.3389/fpls.2022.785804 (PMC8924614; doi:10.3389/fpls.2022.785804)
Supplement: Supplementary file 1 [file Table_1.docx]

**Supplementary Table 1 Correlation analysis for canker severity score versus mean maximum temperature for several months of the growing season**.

| **Month** | **Canker correlation coefficient** |
| --- | --- |
| September temperature | 0.252 |
| October temperature | 0.175 |
| November temperature | 0.189 |
| May temperature | 0.270 |
| June temperature | 0.333 |
| July temperature | 0.261 |
